# Supplementary material for: Loss of Lymphotoxin Alpha-Expressing Memory B Cells Correlates with Metastasis of Human Primary Melanoma
Source: Diagnostics (Basel). 2021 Jul 12;11(7):1238. doi: 10.3390/diagnostics11071238 (PMC8307480; doi:10.3390/diagnostics11071238)
Supplement: Supplementary file 1 [file diagnostics-11-01238-s001.zip › diagnostics-1288381-supplementary.pdf]

Supplementary Material

(A)

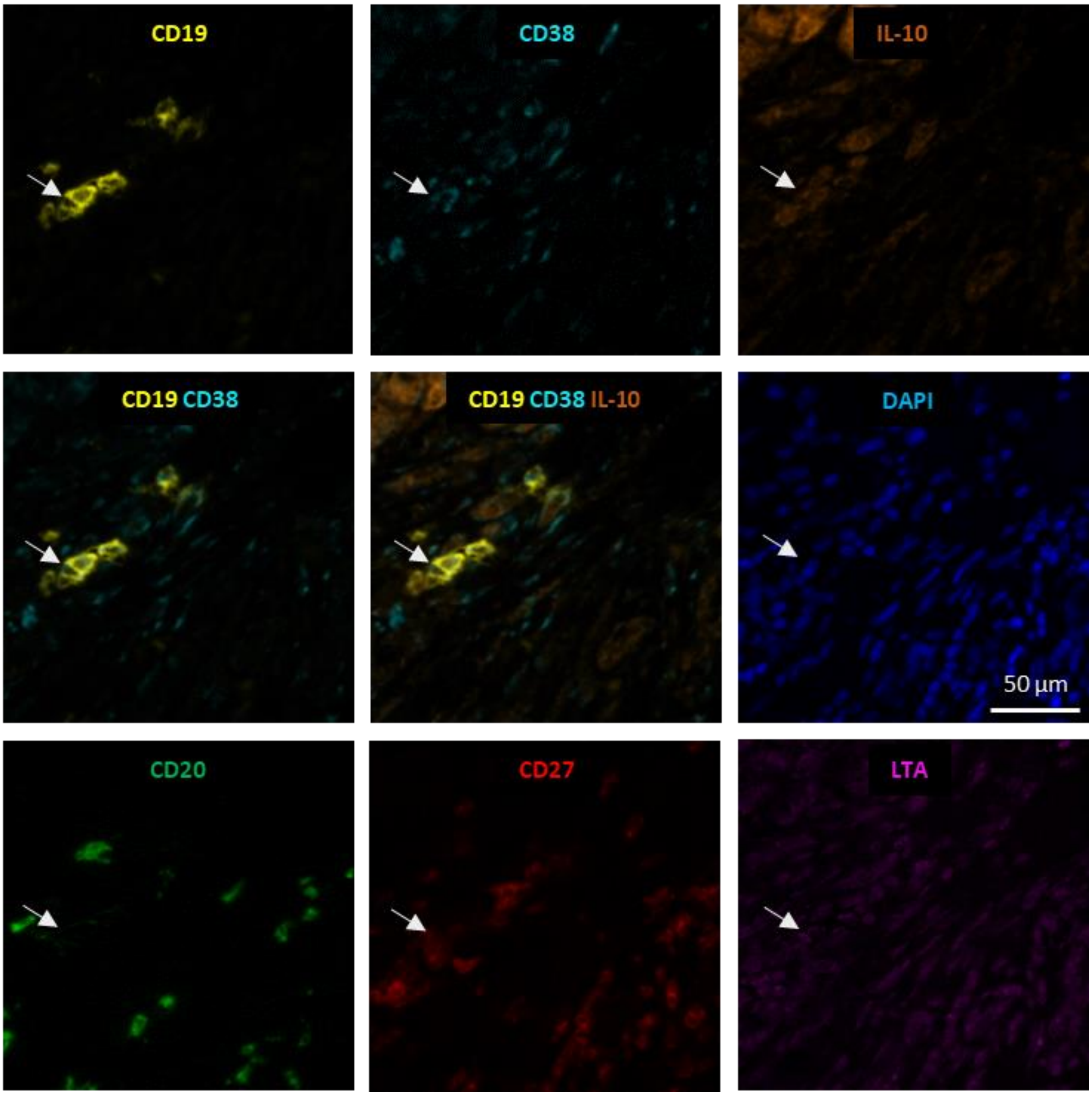

(B)

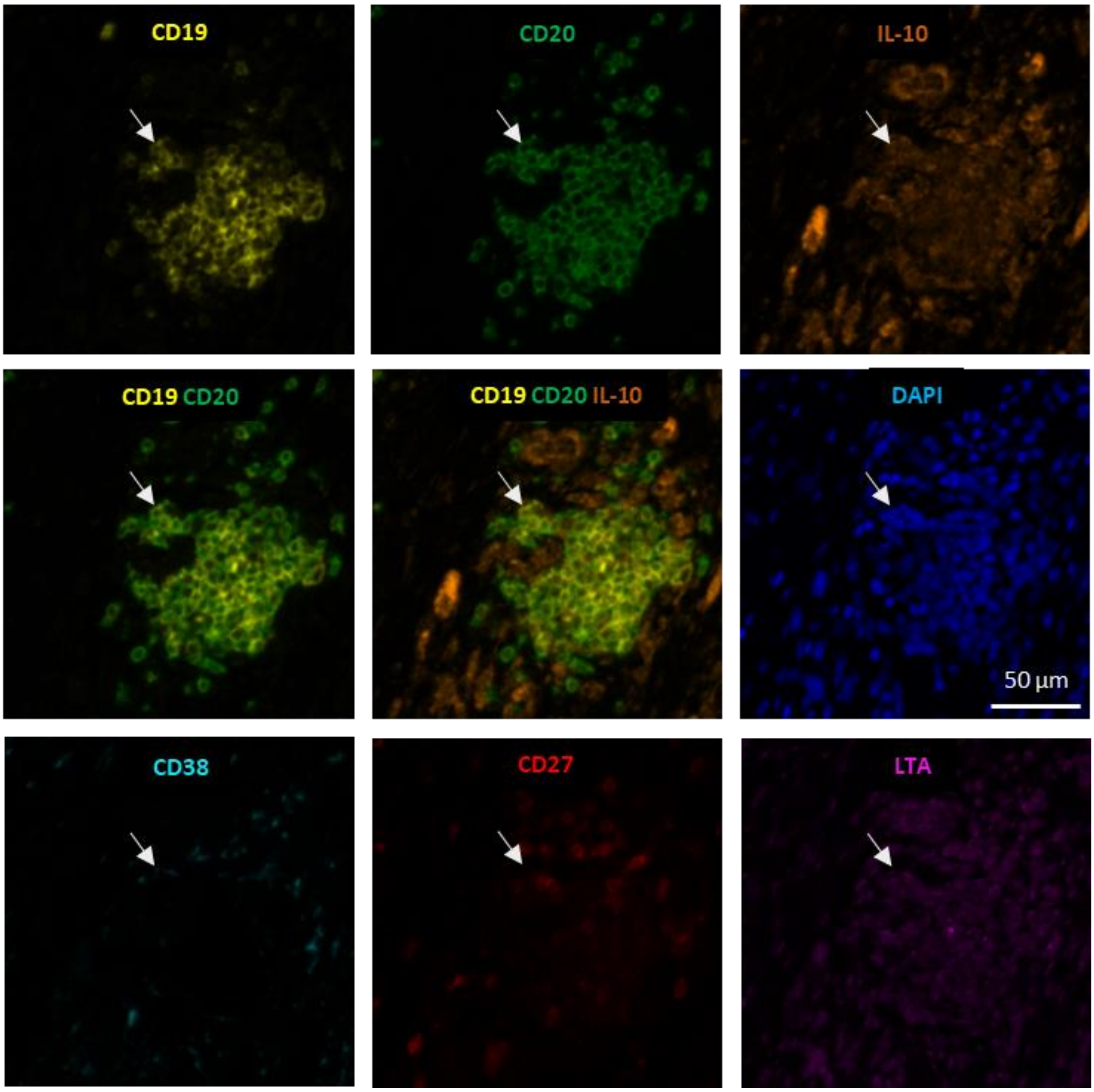
CD19
CD20
IL-10
CD19 CD20
CD19 CD20 IL-10
DAPI
50 µm
CD38
CD27
LTA

(C)

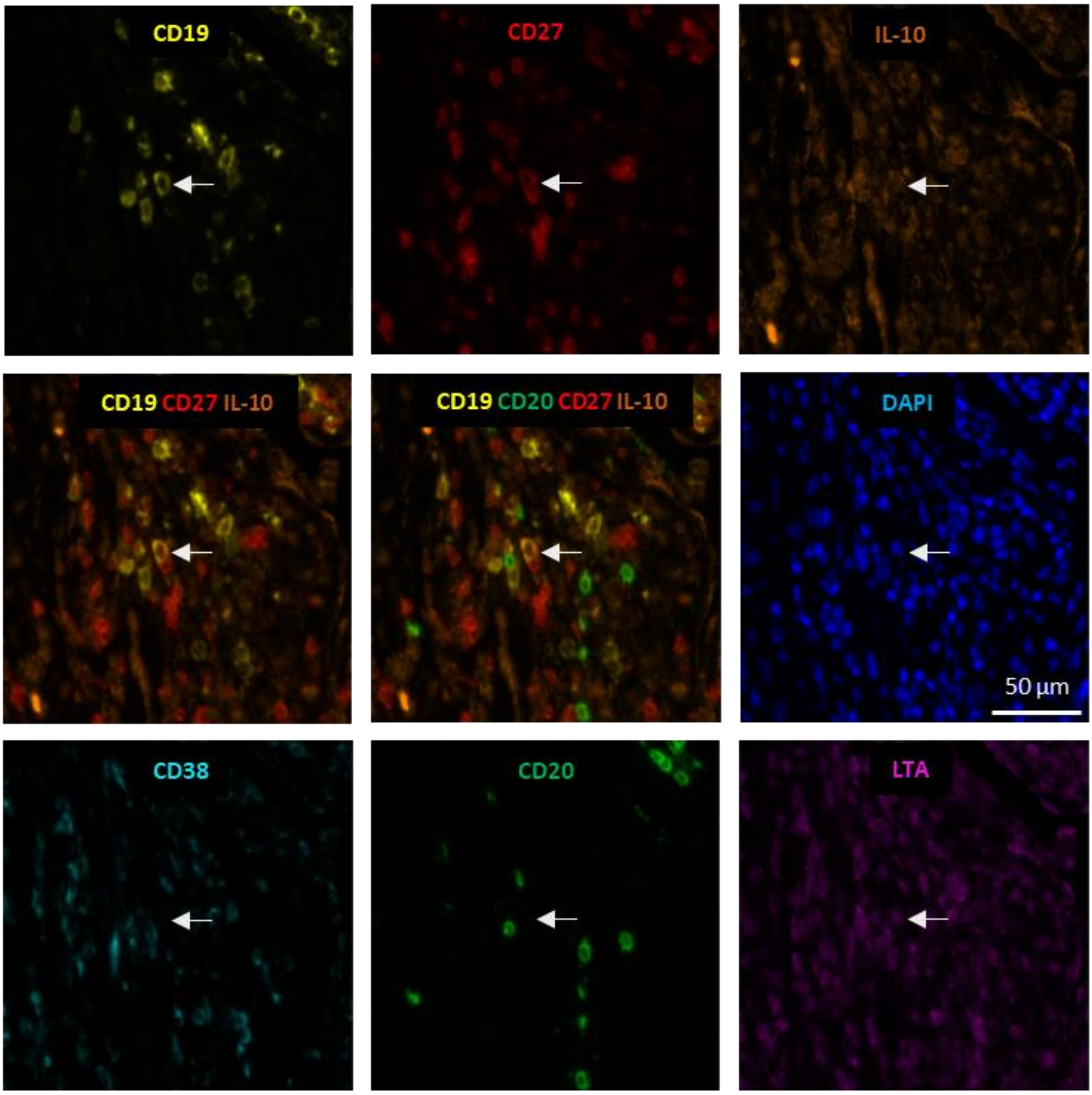

Figure S1. IL-10^{+} B cell subpopulations in human melanoma. Identification of (**A**) IL-10^{+} CD19^{+} CD20^{-} CD27^{-} CD38^{+} antibody secreting cells, (**B**) IL-10^{+} CD19^{+} CD20^{+} CD27^{-} CD38^{-} memory-like and (**C**) IL-10^{+} CD19^{+} CD20^{-} CD27^{+} CD38^{-} activated B cells. Serial images of the same cell for different markers: positive markers are given in the upper row; composite images of positive markers are given in the middle row, together with DAPI nuclear staining in the middle right; negative markers are given in the lower row. Arrows depict the same cell being representative of the respective B cell subpopulation. Scale bars represent 50 µm.

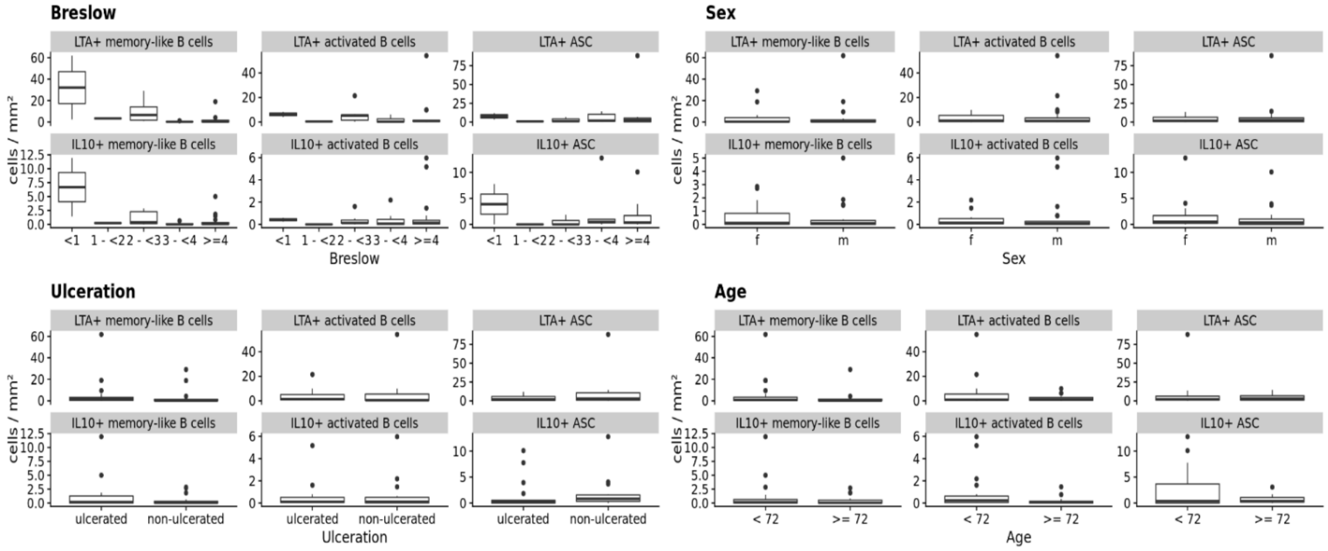

Figure S2. The density (cells/mm²) of LTA⁺ and IL-10⁺ B cell subpopulations in primary human melanomas and their association with another four categorial prognostic clinicopathologic parameters. Box plots comparing primary tumors for increasing Breslow depths (mm), sex, median age (years) and presence of ulceration (from top left to bottom right). In boxplots, lower and upper hinges correspond to the first and third quartiles and center lines to medians. Lower and upper whiskers extend to the largest value within 1.5 times the interquartile range. Outliers are shown as black circles. Association of LTA⁺ memory-like B cell density with Breslow depth ($p=0.05$). ASC = antibody secreting cells.

Table S1. Biopsy sites of melanoma metastases

**No. of patients**	**23**
Skin	4
Lymph node	13
Lung	3
Brain	1
Nerve	1
Kidney	1

Table S2. Comparison of relative frequencies of LTA^+ and IL-10^+ B cell subpopulations in melanoma metastases (Met) to metastasized and non-metastasized primary tumors (PT met and PT non-met)

Cell type	adjusted p-value	lower 95% CI	upper 95% CI	Group1	Group2
LTA+ memory-like B cells	< 0.01	-0.0188	-0.0044	PT met	Met
LTA+ activated B cells	0.01	-0.0379	-0.0075	PT met	Met
IL-10+ ASC	0.16	-0.0171	-0.0011	PT non-met	PT met
LTA+ activated B cells	0.16	-0.0272	-0.0033	PT non-met	Met
IL-10+ ASC	0.16	0.0017	0.0178	PT met	Met
LTA+ memory-like B cells	0.26	0.0004	0.018	PT non-met	PT met
IL-10+ memory-like B cells	0.96	-0.0032	0	PT met	Met
LTA+ ASC	1	-0.0396	0.0015	PT non-met	PT met
IL-10+ activated B cells	1	-0.0031	0.0002	PT non-met	Met
LTA+ ASC	1	-0.0306	0.0028	PT non-met	Met
IL-10+ memory-like B cells	1	-0.0021	0.0007	PT non-met	Met
LTA+ memory-like B cells	1	-0.0121	0.0047	PT non-met	Met
IL-10+ activated B cells	1	-0.003	0.0004	PT met	Met
IL-10+ memory-like B cells	1	-0.0004	0.0018	PT non-met	PT met
LTA+ activated B cells	1	-0.0043	0.0162	PT non-met	PT met
LTA+ ASC	1	-0.0204	0.0343	PT met	Met
IL-10+ activated B cells	1	-0.0008	0.0008	PT non-met	PT met
IL-10+ ASC	1	-0.0007	0.0023	PT non-met	Met
